# Transcriptome dataset of gall-rust infected Sengon (*Falcataria falcata*) seedlings using long-read PCR-cDNA sequencing

**DOI:** 10.1016/j.dib.2023.109919

**Published:** 2023-12-06

**Authors:** Aditya Nugroho, Iskandar Zulkarnaen Siregar, Deden Derajat Matra, Ulfah Juniarti Siregar

**Affiliations:** a Tropical Silviculture Study Program, Graduate School of IPB University, Bogor, Indonesia; b Department of Silviculture, Faculty of Forestry and Environment, IPB University, Bogor, Indonesia; c Department of Agronomy and Horticulture, Faculty of Agriculture, IPB University, Bogor, Indonesia

**Keywords:** Long-read sequencing, Plant defense, Resistance, Sengon, *Uromycladium falcatariae*

## Abstract

Sengon (*Falcataria falcata*) is an economically important legume tree widely cultivated in community forests, especially in Java Island. However, attacks of gall rust disease by *Uromycladium falcatariae* is difficult to manage. Understanding sengon genes expressions when artificially infected with gall rust fungi can help unravel its resistance mechanisms. Total RNA was extracted from sengon seedlings samples inoculated with *U. falcatariae* fungi at 7, 21, and 35 days after inoculation (DAI) and from the control group. Total RNA sequencing was performed using the PCR-cDNA Sequencing protocol (SQK-PCB109) from Oxford Nanopore Technologies. The RNA-Seq obtained varies from 1.3 million to 1.9 million total reads. The assembled full-length transcript was constructed using the RATTLE program, resulting in 21,819 transcripts. The TransDecoder program used to define open reading frames (ORFs) generated 2,342 transcripts, of which 34.15% were 5′prime_partial, 8.15% were 3′prime_partial, 8.5% were internal, and 49.14% were complete. Analysis of differentially expressed genes (DEGs) between resistant and susceptible seedlings, found that 1,013 genes that were up-regulated and 1,130 genes that were down-regulated in the resistant lines. The transcriptome data discussed in this article have been deposited in the DDBJ with accession number DRA015681.

Specifications Table

**Table:** 

Subject	Agricultural Sciences: Forestry
Specific subject area	Transcriptomic study in Forestry
Data format	Raw, Analyzed, Filtered
Type of data	Raw Sequencing reads, Figure, Table
Data collection	RNA sequencing was performed by PCR-cDNA techniques using MinIon apparatus from Nanopore on seedlings leaves treated with artificial inoculation of gall rust fungi and control (non-inoculated) ones. Two groups of seedlings were used, i.e resistant and susceptible ones. They were harvested at 7, 21 and 35 days after inoculation for total RNA extraction.
Data source location	Dramaga, Bogor, West Java, Indonesia (6°33′24.23"S 106°43′33.4"E)
Data accessibility	DDBJ (DNA Data Bank of Japan) with data identification number DRA015681 https://ddbj.nig.ac.jp/resource/sra-submission/DRA015681 Long reads dataset of Sengon (*Falcataria falcata*) infected by gall-rust disease, Mendeley Data, V3, https://doi.org/10.17632/wnfjgzf2kt.3
Related research article	U.J. Siregar, A. Nugroho, H. Shabrina, F. Indriani, A. Damayanti, D.D. Matra. De novo transcriptome assembly data for sengon (*Falcataria moluccana*) trees displaying resistance and susceptibility to boktor stem borers (*Xystrocera festiva* Pascoe). BMC Res Notes. 14 (2021) 1-4. DOI: 10.1186/s13104-021-05675-9

## 1 Value of the Data

• The data provide *F. falcata* seedling transcriptome reference using Oxford Nanopore Technologies of PCR-cDNA long-read sequencing after gall rust artificial inoculation
• The presented dataset could help to explain the mechanisms of *F. falcata* resistance to gall rust disease
• The data is beneficial to researchers involved in identifying genes that show differential expression during gall rust infection process and can be used to create genetic markers that will serve as a valuable tool for improvement of gall rust-resistant *F. falcata* trees.

## 2 Data Description

**Fig. 1:**
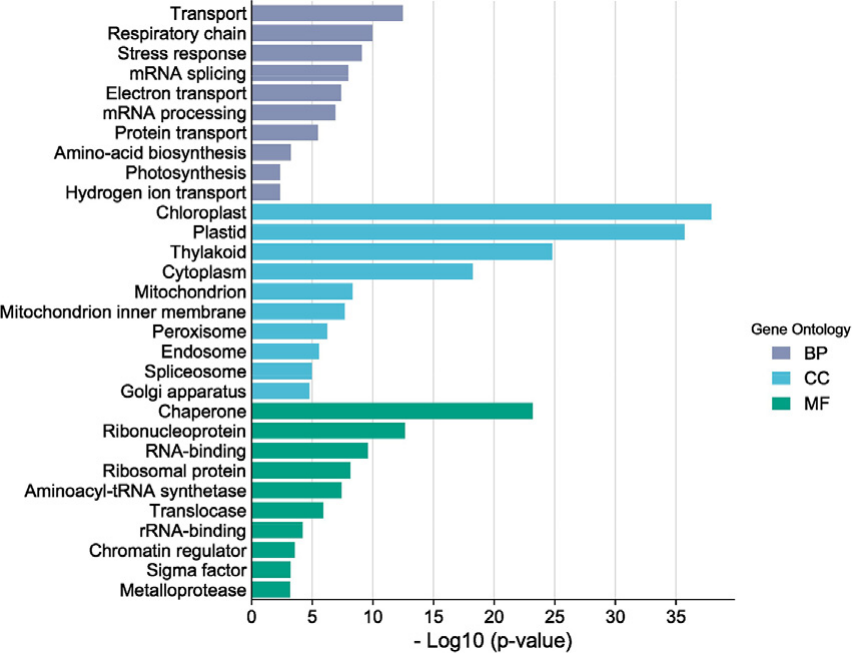
Gene Ontology (GO) classification of *Falcataria falcata* transcripts. note: BP = biological process, CC = cellular component, MF = molecular function

**Fig. 2:**
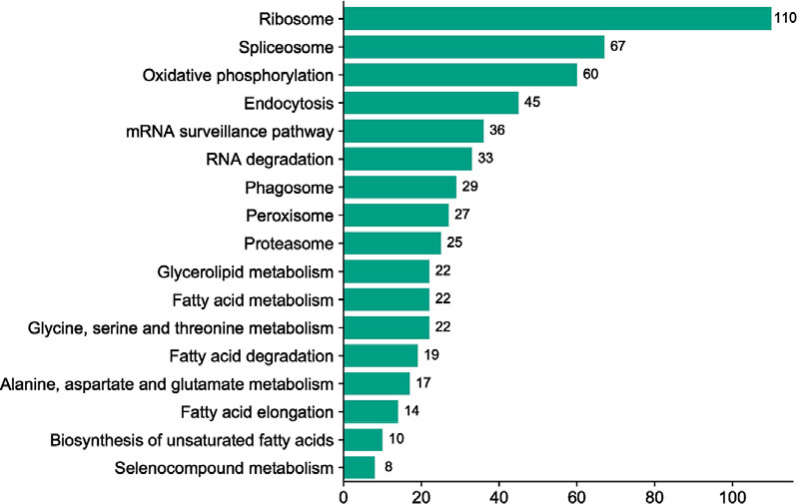
KEGG pathway of *Falcataria falcata* transcripts.

**Fig. 3:**
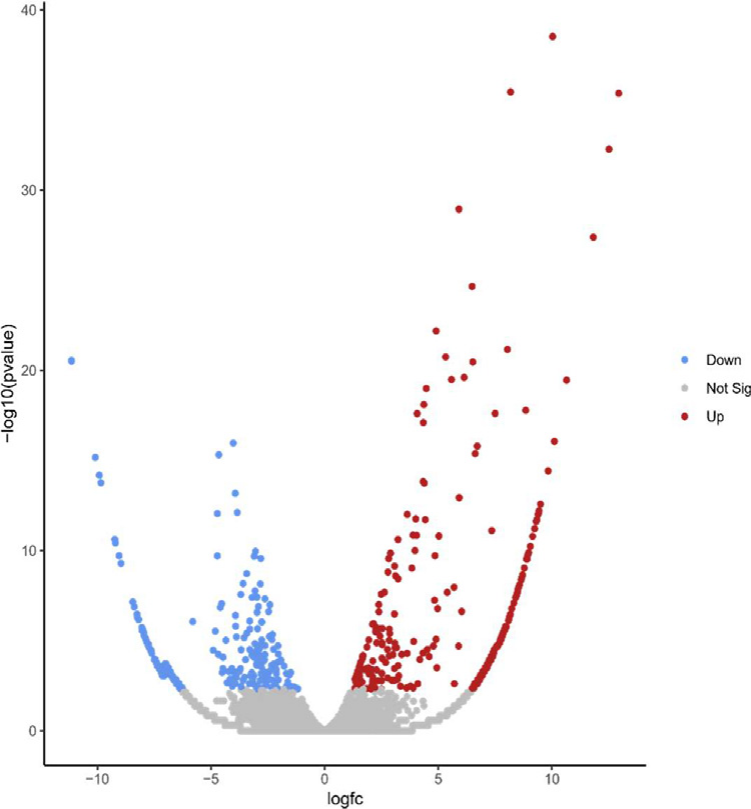
Volcano plot of DEGs from resistant and susceptible *Falcataria falcata* seedlings.

A total of eight RNA libraries consisted of resistant control, resistant 7 DAI (Day After Inoculation), resistant 21 DAI, resistant 35 DAI, susceptible control, as well as susceptible 7 DAI, susceptible 21 DAI, and susceptible 35 DAI were prepared and sequenced by PCR-cDNA long-read sequencing using MinION apparatus from Oxford Nanopore Technologies. Total RNA was extracted from each seedling leaves using Plant Total RNA Mini Kit (Geneaid) according to the manufacturer protocol. The raw data generated during sequencing were then preprocessed using Phychopper [1] and Cutadapt [2] to remove SSP (strand-switching primer), VNP (oligo-dT_30_VN), and polyA tails present in the reads. The raw and preprocessed data are shown in Table 1. De novo assembly was then performed on the clean reads data using RATTLE [3], [4], [5], which resulted in 21,819 transcripts (Table 2). The assembled data transcripts were then annotated using BLAST+ v.2.7.1 [6]. With a filtered-UNIPROT database and processed with Blast2go 6.0 software [7]. The TransDecoder program v.5.5.0 with default parameters [8,9] was then used to define open reading frames (ORFs) and generated 2,342 transcripts, of which 34.15% were 5′prime_partial, 8.15% were 3′prime_partial, 8.5% were internal, and 49.14% were complete (Table 2). An overview of the Gene Ontology (GO) classification generated from *F. falcata* is shown in Fig. 1. Gene Ontology is divided into three classifications, namely biological process, molecular function, and cellular component. The gene ontology that has the highest -log(10) value in each classification is transport for biological process, chaperone for molecular component, and chloroplast for cellular component. Fig. 2 shows the 17 KEGG pathways identified in *F. falcata*. Differentially Expressed Genes (DEGs) analysis on resistant vs susceptible seedlings using R v.4.1.0 software with edgeR v.3.34.0 package showed that there were 1,013 up-regulated genes and 1,130 down-regulated genes in the resistant lines (Fig. 3). The top ten up-regulated and down-regulated genes associated with defense responses to biotic stress in resistant vs susceptible *F. falcata* seedlings is shown in Table 3.

**Table 1:** Summary of raw and clean reads statistics.

Sample	RA	RB	RC	RD	SA	SB	SC	SD
Raw Reads								
Total Reads	1,802,408	1,325,854	1,997,244	1,470,662	1,876,307	1,874,318	1,736,895	1,889,164
Average (bp)	100.9	60	119.4	121.2	69.2	100	80.4	100.5
Largest (bp)	3,030	3,743	4,036	27,158	3,036	3,819	2,346	3,822
N50 (bp)	146	140	179	184	98	153	110	156
Cleaned Reads								
Total Reads	1,301,804	835,725	1,562,232	1,111,882	1,130,027	1,450,444	1,324,635	1,498,712
Average (bp)	110.9	107.5	133.6	130	89.5	112.9	92.7	113.3
Largest (bp)	2,789	2,923	3,137	11,823	2,736	2,639	2,343	2,482
N50 (bp)	146	139	175	175	103	152	114	156

note: RA = resistant control; RB = resistant 7 DAI; RC = resistant 21 DAI; RD = resistant 35 DAI; SA = susceptible control; SB = susceptible 7 DAI; SC = susceptible 21 DAI; SD = susceptible 35 DAI

**Table 2:** Summary of *de novo* transcriptome assembly and open reading frames (ORFs) prediction characteristics.

Features	Numbers
Total transcripts	21,819
bases total (bp)	11,779,495
Average (bp)	539.9
Length range (bp)	147 – 11,755
N50 (bp)	615
ORF transcripts	2,342
- 5′prime_partial	800 (34.15%)
- 3′prime_partial	191 (8.15%)
- Internal	200 (8.5%)
- Complete	1,151 (49.14%)

**Table 3:** Top six each of up-regulated and down-regulated genes associated with defense responses to biotic stress in resistant vs susceptible *Falcataria falcata* seedlings.

Transcript name	log Fold Change (logFC)	False Discovery Rate (FDR)	Uniprot accessions	Gene description
Transcript_cluster_19027	7.93519	8.18E-05	Q9LMA1	Probable flavin-containing monooxygenase 1
Transcript_cluster_20880	7.17828	3.44E-03	Q3EDG5	Protein-tyrosine sulfotransferase
Transcript_cluster_14591	5.28438	6.88E-01	Q42602	Cytochrome P450 89A2
Transcript_cluster_9038	4.78636	5.00E-02	O81825	Probable disease resistance protein At4g27220
Transcript_cluster_20946	2.52912	3.66E-04	Q9LMA1	Probable flavin-containing monooxygenase 1
Transcript_cluster_21620	0.53773	4.50E-04	O64782	G-type lectin S-receptor-like serine/threonine-protein kinase
Transcript_cluster_18716	-6.21051	6.71E-02	O04153	Calreticulin-3
Transcript_cluster_12197	-6.22086	6.71E-02	Q9LHA7	Peroxidase 31
Transcript_cluster_21206	-6.86276	7.63E-03	P27323	Heat shock protein 90-1
Transcript_cluster_20677	-7.47480	2.21E-03	Q9SCP5	UDP-glycosyltransferase 73C7
Transcript_cluster_20770	-8.09446	2.83E-05	Q9SV43	Patatin-like protein 7
Transcript_cluster_16372	-5.42326	2.32E-03	O04379	Protein argonaute 1
Transcript_cluster_5379	-2.39366	3.68E-04	Q9SUR9	Protein SGT1 homolog A

## 3 Experimental Design, Materials and Methods

### 3.1 Plant Materials

**Fig. 4:**
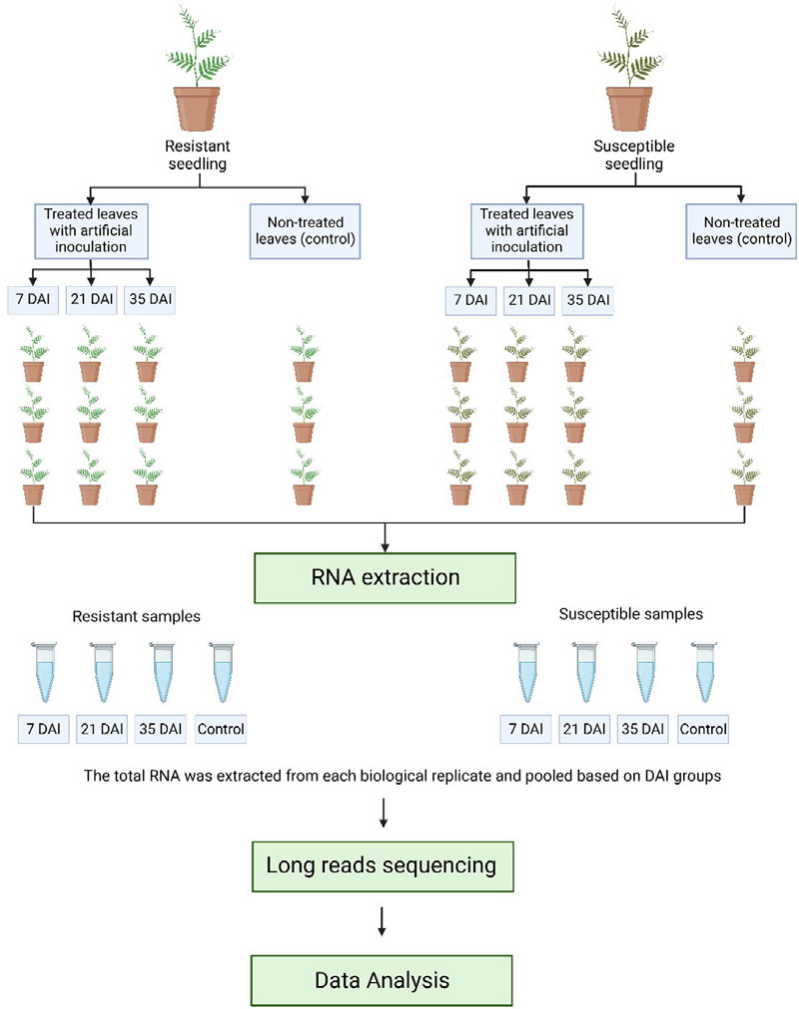
Experimental design for long reads sequencing (Created with BioRender).

Two groups of two-month-old sengon seedlings, i.e resistant and susceptible lines (Fig. 4) were used for artificial inoculation with *U. falcatariae* fungi [10] with control (non-inoculated ones) from each group. All seedlings were grown in the greenhouse of the Faculty of Forestry and Environment, IPB University, Indonesia (6°33*'*24.23″S 106°43*'*33.4″E). The leaf samples were collected from seedlings on 7, 21, and 35 days after inoculation (DAI) and from the control group (Fig. 4). Three seedlings were used as biological replicates.

### 3.2 Total RNA Extraction and Sequencing

The RNA was extracted from leaves using the Plant Total RNA Mini Kit (Geneaid) according to the manufacturer protocol. The total RNA was extracted from three biological replicate and pooled based on DAI groups (Fig. 4). The quality and quantity of the RNA were checked using the Nanophotometer NP-80 (Implen) and the Qubit™ RNA Broad Range (BR) assay on the Qubit® Fluorometer (Invitrogen). The extracted RNA was then used for RNA sequencing using PCR-cDNA Barcoding-SQK-PCB109 (PCB_9092_v109_revB_10Oct2019). The sequencing process was performed on a Flow Cell R9.4.1 (FLO-MIN106D) using the MinION Mk1B.

### 3.3 Transcriptome Assembly and Mapping

The raw fast5 files were base called using Guppy v6.1.3+cc1d765d3 with default parameters [11]. Then, the data was preprocessed according to the protocol described at https://github.com/felixgrunberger/microbepore, and using NanoStat v1.2.1 to evaluate the quality and statistics of the reads [12]. Reads were then cleaned and trimmed using phychopper v2.5.0 [1]. All remaining polyA tails, VNP, and SSP adapters were removed using Cutadapt v3.2 [2]. Finally, de novo assembly was performed using the RATTLE program [3] with the resulting clean full-length transcripts. [7]. In addition, the open reading frames (ORFs) within the transcripts were predicted using TransDecoder v.5.5.0 (https://github.com/TransDecoder/TransDecoder, accessed on 1 November 2022) with the default parameters [8,9]. Cleaned-reads were also mapped back to full-length transcripts using minimap2 v.2.26 [13].

### 3.4 Functional Annotation

The assembled transcripts were annotated with the BLAST+ v.2.7.1 [6] using a filtered UNIPROT database (Viridiplantae TaxID: 2759, downloaded October 19, 2021) with a cut-off threshold of 10^−5^. The resulting blast output was then processed using Blast2Go v.6.0 software to obtain functional annotations for Gene Ontology and KEGG pathways. DEGs analysis was performed using R v.4.1.0 software with edgeR v.3.34.0 package to estimate the expression levels of all transcripts [14]. The results of the DEGs analysis are visualized with volcano plot using the tools available in the usegalaxy (https://usegalaxy.eu/) web interface with logFC ≥ 2. Gene ontology and KEGG pathways of DEGs from resistant and susceptible seedlings were performed using the DAVID 2021 beta (https://david.ncifcrf.gov/tools.jsp) web interface [15,16].

## Limitations

This study used seedlings derived from the seeds of resistant and susceptible parent trees that cross-pollinate in nature. In addition, due to limitations in obtaining good quality total RNA without degradation and fragmentation during library construction, sample pooling was carried out.

## Ethics Statement

No human subjects, animal experiments, or any data collected from social media platforms were performed to obtain the data

## CRediT authorship contribution statement

**Aditya Nugroho:** Investigation, Formal analysis, Visualization, Writing – original draft. **Iskandar Zulkarnaen Siregar:** Supervision, Writing – review & editing. **Deden Derajat Matra:** Supervision, Data curation, Validation, Writing – review & editing. **Ulfah Juniarti Siregar:** Conceptualization, Funding acquisition, Supervision, Writing – review & editing.

## Data Availability

Research Data (Original data) (Mendeley Data).
